# Sparse Depth-Guided Image Enhancement Using Incremental GP with Informative Point Selection

**DOI:** 10.3390/s23031212

**Published:** 2023-01-20

**Authors:** Geonmo Yang, Juhui Lee, Ayoung Kim, Younggun Cho

**Affiliations:** 1 Department of Electrical and Computer Engineering, Inha University, Incheon 22212, Republic of Korea; 2 Department of Mechanical Engineering, Seoul National University, Seoul 01811, Republic of Korea

**Keywords:** image enhancement, dehazing, Gaussian Process, underwater

## Abstract

We propose an online dehazing method with sparse depth priors using an incremental Gaussian Process (iGP). Conventional approaches focus on achieving single image dehazing by using multiple channels. In many robotics platforms, range measurements are directly available, except in a sparse form. This paper exploits direct and possibly sparse depth data in order to achieve efficient and effective dehazing that works for both color and grayscale images. The proposed algorithm is not limited to the channel information and works equally well for both color and gray images. However, efficient depth map estimations (from sparse depth priors) are additionally required. This paper focuses on a highly sparse depth prior for online dehazing. For efficient dehazing, we adopted iGP for incremental depth map estimation and dehazing. Incremental selection of the depth prior was conducted in an information-theoretic way by evaluating mutual information (MI) and other information-based metrics. As per updates, only the most informative depth prior was added, and haze-free images were reconstructed from the atmospheric scattering model with incrementally estimated depth. The proposed method was validated using different scenarios, color images under synthetic fog, real color, and grayscale haze indoors, outdoors, and underwater scenes.

## 1. Introduction

In field robotics, visual recognition is the main idea behind robot navigation and task performance. However, images can be degraded by poor atmospheric conditions or being underwater, which raises problems with visual recognition using cameras, such as haze effects, contrast loss, and color distortion. Visible enhancement of these degraded images is important for many robotic applications, such as SLAM (simultaneous localization and mapping) [1,2], object recognition and grasping [3], and underwater robotics [4,5]. Many previous studies of dehazing [6,7,8,9,10] are biased toward the visibility of restoring a single image for monitoring. However, robot navigation with real hazard images causes false localization and mapping. It eventually leads to crucial problems on the real self-driving ability of robots. Generally, degraded images are generated by light absorption, scattering by particles, water droplets, and many other external factors. Moreover, images captured in a fiery, smoke-laden room or underwater are extremely damaged by very dense turbidity and are limited to short ranges.

In the early stages of image dehazing for atmospheric images, logarithmic image processing and contrast enhancement methods were widely applied to achieve better visibility of scenes. There are many related works on turbid atmosphere conditions [6,7,11]. Effective single image dehazing methods used Independent Component Analysis (ICA), which was introduced in [6]. ICA is the first breakthrough in haze removal methods using single images. However, it does not work well on grayscale images and requires lots of computational time. He [7] demonstrated single image dehazing using Dark Channel Prior (DCP). This idea is related to a strong prior that at least one of the channels (i.e., of R, G, and B) of each pixel is low in haze-free images. DCP-based dehazing is effective and simple, but the channel prior is weak for sky regions and underwater images. Moreover, it is hard to operate in real time because of its soft-matting technique, which requires a lot of computational time. He [11] proposed guided filters for time-effectiveness, but a weakness is admitted for sky regions and underwater.

Dehazing methods underwater have also been developed to enhance visibility for underwater exploration. Underwater single-image dehazing methods using wavelength-dependent attenuation of light in water were developed in [9]. They calculate the depth prior from the strong difference in attenuation between color channels, then use it to recover the scene radiance by Markov random field (MRF). Ancuti et al. [10] proposed multi-scale fusion-based image dehazing with two generated inputs. It is time effective for underwater images; however, it cannot construct relative depth or a transmission map. Cho et al. [12] utilized the Gaussian process for depth estimation, but the performance was limited and did not consider the information level of incoming depth information. Berman et al. [13] proposed a haze line-based approach to estimate ambient color and transmission within color-biased environments. There are also studies that have improved image information using sparse depth obtained from robot platforms. Babaee and Negahdaripour [14] used sparse sonar depth in an underwater environment for hybrid dehazing methods by fusing optical images and acoustic images. With previous research on sensor fusion, Babaee and Negahdaripour [14] matched the partial depth of imaging sonar to optical images and applied MRF with the intensity of optic images and acoustic images to estimate dense depth maps. This can predict a real-scale depth map from sonar images and reconstruct dehazed images in strong haze optical images. However, image enhancement works weaker and the MRF-based slows it down. Cho and Kim [15] exploits Doppler velocity log (DVL) sparse depth to enhance underwater images. Because planar scenes should be assumed, there are difficulties when applying to non-planar situations.

As research related to deep learning increases, dehazing research on the underwater environment has also increased. These studies attempted to restore the image itself using the network structure of CNN (convolution neural network), GAN (generative adversarial network), or ViT (vision transformer). In [16], the authors performed image restoration with a clean underwater image using a CNN network structure for underwater images (UWCNN). This paper introduced the underwater image synthesis method with various water types and turbidity and utilized the structure similarity error to preserve the original textures of images. Wang et al. [17] proposed CNN-based image enhancement incorporating both RGB and HSV color space images. This method utilizes HSV color transform in the network to compensate for the weakness of the RGB color space that is insensitive to the luminosity and saturation of the image. This method can remove color cast and include detailed information. Fabbri et al. [18] performed image enhancement using GAN. CycleGAN was used to form a ground truth and distorted image pair. Given a distorted image, CycleGAN converts it as if it came from the domain of an undistorted image. The image pair created is used for image reconstruction. FUnIE-GAN, based on the conditional GAN network, was also introduced in [19]. The loss function of the FUnIE-GAN evaluates the perception quality of images, such as global similarity, content, local text, and style. Image enhancement can also be applied to both paired and unpaired images.

There are several studies on aerial image dehazing with deep learning-based approaches. Li et al. [20] proposed light-weight CNN to convert haze images to clean images. Song et al. [21] presented DehazeFormer, which is a combination of the Swin Transformer and U-Net with several critical modifications, including the normalization layer, activation function, and spatial information aggregation scheme. citetqin2020ffa proposed a feature fusion attention network using local residual learning and feature attention modules, which give more weight to more important features. Liu et al. [22] introduced a CNN structure for a single image dehazing. To alleviate the bottleneck problem, it applied an attention-based multi-scale estimation on the grid network.

From previous methods, we can deduct several objectives of dehazing issues for robotics problems. First, the method should run in real time and ’learn’ data online. Second, the algorithm should work regardless of the channels. The last objective is involves the estimation of the normalized depths of images. Depth information is important and necessary not only for dehazing but also for mapping issues.

In this paper, we propose an online image dehazing method with partial and sparse depth data using iGP depth estimation. We achieved a general method for both color and grayscale images with minimum user parameters. To estimate the reliable depth, we used partial depth information with low-level fusion. As mentioned in [14], a dense depth map is needed to reconstruct haze-free images in a strong turbid medium. It is easy to detect the partial depth data of optical images by simple sensor fusion. In comparison with [14], the proposed method only requires low level fusion with any range sensors. Our proposed method can choose the most informative input data points, which cover the vulnerable region of estimated depth. We automatically chose dehazing parameters, such as airlight and transmission. The evaluation was performed with other previous methods by comparing qualitative results (visibility) and quantitative results (feature matching is presented). In summary, the contributions to our methods are as follows:

- Online depth learning using iGP;
- Information theoretic data point selection;
- Quantitative measures for dehazing qualities based on information metrics;
- Independence of the channel number (both color and gray images).

This paper proceeds as follows. In Section 2, we introduce a general atmospheric scattering model for dehazing. The method is described in Section 3 and Section 4. We present experimental results in Section 5.

## 2. Atmospheric Scattering Model

We first describe the atmospheric scattering model used in this paper. As introduced in [23], the haze image I(x) is modeled as a weighted sum of a haze-free image (scene radiance) J and global airlight A, while the weight between the two is determined by medium transmission t(x), as in (1)

(1) I(x)=J(x)t(x)+A(1−t(x))

Transmission t(x) controls color attenuation due to the scattering medium, and is often modeled as a function of depth at a pixel point x (2). In this transmission function, the level of attenuation is determined by an attenuation coefficient (β), which reflects the thickness of the haze.

(2) t(x)=exp(−βd(x))

This paper focuses on estimating a transmission map from range sensor measurements for fast dehazing. Once a transmission map is obtained, the dehazing process is fairly intuitive using the atmospheric scattering model. We used a dehazing method introduced in [23], using normalized depth that has values between 0 and 1 for accurate prediction and estimation generality. Since the depth is normalized, the proposed method can be applied to all general depth sensors. From this normalized depth, an attenuation coefficient β is bounded and easily estimated by the brightness rate of pixels.

## 3. Incremental Gaussian Process

### 3.1. Gaussian Process Regression

For the transmission map t(x) presented in the previous section, we exploit Gaussian Process (GP) using highly sparse data. GP is known as a powerful learning model especially when data are spatially related and partial. GP learns a distribution over the space of any finite number of data with a joint Gaussian distribution. GP is defined by a mean function μ(x) and covariance function $k(x,x^{\prime })$ where x and $x^{\prime }$ are data samples. During training, GP learns the correlation between data via a covariance function and predicts not only the mean but also the variance.

To predict the output for the target data, we assume joint Gaussian probability between test data $x^{\prime }$ and training data $X=\{x_{1:n}\}$. In the training phase, we construct Gram matrix K(X,X)=K using the defined covariance function so that the Gram matrix *K* contains characteristics of the training samples. The prediction for a test input x involves obtaining a conditional Gaussian distribution given the training inputs $p\left(f_{*}|x_{*},X,y\right)=N\left(\mu_{*},\Sigma_{*}\right)$, whereas the estimated mean and covariance are given as below; y is a training output, $\sigma_{n}$ is observation noise, and *I* represents the identity matrix.

(3) $$\begin{array}{ccc} \mu_{*} & = & k{\left(x_{*},X\right)}^{⊤}{\left(K+\sigma_{n}^{2}I\right)}^{-1}y\\ \Sigma_{*} & = & k\left(x_{*},x_{*}\right)-k{\left(x_{*},X\right)}^{⊤}{\left(K+\sigma_{n}^{2}I\right)}^{-1}k\left(x_{*},X\right) \end{array}$$

### 3.2. iGP Kernel Function

The selection of kernel functions and hyperparameters affect the GP prediction performance. We constructed kernels using both stationary and non-stationary kernel functions. For stationary kernel, we used a squared exponential (SE) kernel, which is one of the most popular kernel functions with hyperparameters θ=(σ,l) where σ is a standard deviation of additional Gaussian noise and *I* is an identity matrix that is the same size as *K*.

(4) $$k\left(x,x^{\prime }\right)=\sigma^{2}\exp\left(\frac{-\left|\right|\left(x-x^{\prime }\right){\left|\right|}^{2}}{2l^{2}}\right)$$

For the SE kernel, hyperparameters are optimized by maximizing the marginal log-likelihood. To incorporate sudden change in spatial distribution, we additionally include a non-stationary kernel function. Although hyperparameter optimization allows short-term characteristics, a single kernel cannot cover both disconnected short-term and long-term tendencies simultaneously. The design of the kernel is then capable of covering both short-term and long-term characteristics. We chose a mixture of Neural Network (NN) and SE kernels as in (5), where $\sigma_{SE}$ and $l_{SE}$ are hyperparameters of the SE kernel, $\tilde{x}=\left(1,x_{1},\cdots ,x_{d}\right)$ is an augmented input vector, and $\Sigma =\mathrm{diag}(\sigma_{0}^{2},\sigma^{2})$.

(5) $$\begin{array}{ccc} k(x,x^{\prime }) & =\underset{SE}{\underset{︸}{\sigma_{SE}^{2}\exp\left(-\frac{\left|\right|\left(x-x^{\prime }\right){\left|\right|}^{2}}{2l_{SE}^{2}}\right)}}\\ & +\underset{NN}{\underset{︸}{\frac{2}{\pi }\sin^{-1}\left(\frac{2{\tilde{x}}^{⊤}\Sigma {\tilde{x}}^{\prime }}{\sqrt{\left(1+{\tilde{x}}^{⊤}\Sigma {\tilde{x}}^{\prime }\right)\left(1+\tilde{x}\Sigma {\tilde{x}}^{\prime ⊤}\right)}}\right)}} & \end{array}$$

### 3.3. iGP with QR Decomposition

To achieve an efficient GP capable of online processing, we constructed iGP using QR decomposition following [24]. By applying the Givens rotational-based QR decomposition, the inverse of matrix ($K+\sigma^{2}I$) is computed incrementally. If decomposition matrices of $K+\sigma^{2}I$ are $Q_{K+\sigma^{2}I}$ and $R_{K+\sigma^{2}I}$ (i.e., $K+\sigma^{2}I=Q_{K+\sigma^{2}I}R_{K+\sigma^{2}I}$), then the inverse of $K+\sigma^{2}I$ can be obtained by following the simple matrix calculation.

(6) $${\left(K+\sigma^{2}I\right)}^{-1}=R_{K+\sigma^{2}I}\backslash Q_{K+\sigma^{2}I}^{⊤}$$

QR decomposition with Givens rotation has two advantages, as follows. First, it allows practical convenience of QR decomposition-based matrix inversion. The Cholesky decomposition, which is popularly used for inverse needs, has a strong prior that the matrix should be positive semidefinite (PSD). Theoretically, Gram matrix *K* is always PSD. However, the matrix rarely deviates from PSD due to numerical issues or inappropriate hyperparameter selection. To handle this issue, Senelson [25] introduced a ‘jitter’ term for numerical stability of the Cholesky decomposition. The QR decomposition is beneficial as it does not require an additional term. Secondly, we can only update newly added measurements without repeatedly recalculating previous inputs, and this makes the algorithm substantially more effective in practice.

**Figure 1:**
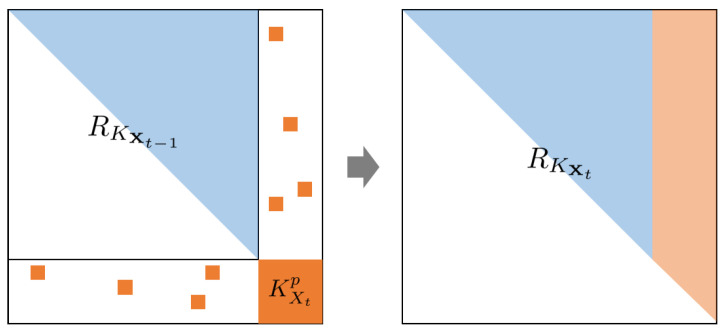
QR decomposition illustration. (**Left**) Covariances of newly added inputs are colored orange while the $R_{K_{X_{t-1}}}$ matrix is the *R* matrix of QR decomposition of the previous update. (**Right**) Updated $R_{K_{X_{t-1}}}$ matrix with added new inputs. We drop $+\sigma^{2}I$ on each Gram matrix for the simple notation.

This incremental update is illustrated in Figure 1 as four phases. $K_{X}$ stands for the Gram matrix of previous inputs and is written as $K_{XX}$. $K_{X^{t}}$ means the Gram matrix of newly added inputs at the *t* update and is written as $K_{X^{t}X^{t}}$. Covariances of newly added inputs and $R_{X}$ of the previous updates are given in the left figure. To obtain the $R_{K_{X}+\sigma^{2}I}$ matrix, which is upper-triangular, we simply apply Givens rotations on new inputs in the $K_{X^{t}X}$ submatrix and $K_{X^{t}}+\sigma^{2}I$ itself. Lastly, updated $R_{K_{X_{t}}}$ from new training data was obtained in an upper triangle matrix (such as the right figure).

## 4. Information Enhanced Online Dehazing

In many robotics applications that use range measurements, a very sparse range prior is often available. For instance, Light Detection and Ranging (LIDAR) for the aerial/ground platform may be sparse, and DVL provides useful four range measurements from beams together with the velocity of an underwater vehicle. In this section, we discuss intelligent training point selections using information measures. We propose simultaneous dense depth estimations and dehazing with information-enhanced iGP. For efficient depth estimation with iGP, selecting the training points needs to be done intelligently in order to keep the accumulated training inputs minimal.

Two types of MI are introduced. First, we measured MI between previous training inputs and newly obtained inputs, and chose more informative points among new input candidates. Secondly, we measured the MI of the GP model that appeared in [26]; this revealed the information levels in the candidate points. In this work, we used the latter measure when (*i*) verifying training inputs and (*ii*) deciding on a stop criterion.

Although the proposed method can be applied to any type of range measurement, we illustrate our method assuming one of the common sensor configurations in [27] with incrementally updated vertical depth points for explanations. Note that the proposed method is applicable to general range data.

### 4.1. Active Measurement Selection

Let $X=\{x_{1},\cdots ,x_{n}\}$ be the *n* existing training inputs until *t* step, and $X^{t}=\left\{x_{1}^{t},\cdots ,x_{m}^{t}\right\}$ be the *m* newly added training inputs at the current time step *t*. A single input datum x is defined as a five-dimensional vector x=[u,v,r,g,b] for a color image and a three-dimensional vector x=[u,v,i] for a grayscale image. Each vector consists of a pixel location (u,v), and three channels (r,g,b) or an intensity channel (*i*).

For informative training data point selections are essential for deciding which point is the best to cover the weak estimation region of previous training. We find the necessity of a new metric for choosing the best point. There are two objectives of the metric, the first one is that it is computationally efficient and the second is that it produces meaningful information between inputs. One of the key features of GP is estimation variance that can be used for data selection. Intuitively, test inputs that are most far from the training inputs have the largest uncertainties, meaning that new training inputs should be uncorrelated with previously selected inputs. By the characteristics of GP, each input point constructs the multivariate Gaussian distribution; therefore, we can use the MI of the Gaussian distribution. The MI of two input points, $x_{i}$ and $x_{j}$, can be defined as below.

(7) $$I\left(x_{i},x_{j}\right)=-\frac{1}{2}\ln\left(1-{\left(\frac{\sigma_{x_{i}x_{j}}}{\sigma_{x_{i}}\sigma_{x_{j}}}\right)}^{2}\right)$$

**Figure 2:**
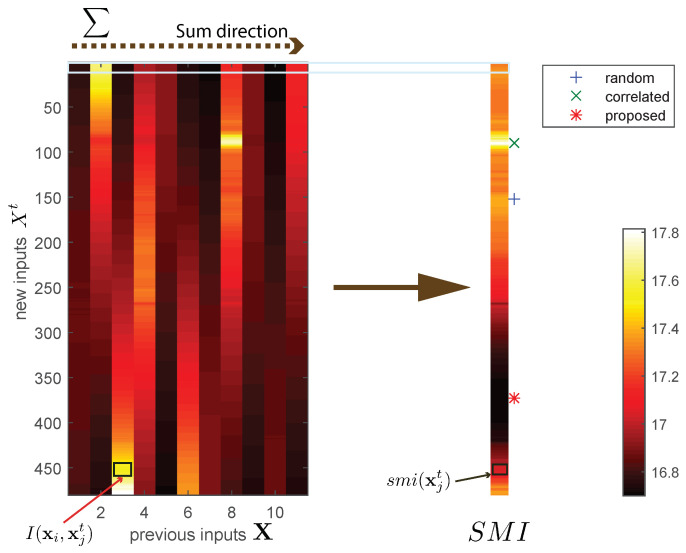
MI matrix and corresponding SMI visualization. Left matrix plots MI between all previous inputs X (horizontal axis) and new inputs X (vertical axis). Middle figure bar indicates the SMI value for each row. We compare three types of point selections (random, correlated, and proposed).

As shown in (7), MI is a function of the covariance values from the Gram matrix, i.e., the ii element ($\sigma_{x_{i}}$) and ij element ($\sigma_{x_{i}x_{j}}$) of the Gram matrix up to time *t*. Given the current Gram matrix (n×n) and *m* newly added training points, the naive operation of the Gram matrix handles the whole (m+n)×(m+n) matrix, which can be significantly inefficient. Our finding is that the correlation between the existing and newly added inputs has a major impact. This correlation is described in Figure 2 as the m×n matrix. To evaluate the information for each newly added data point, we introduce Sum of Mutual Information (SMI) for each candidate, to sum each row (i.e., summation of MI between the *j*th candidate of all other previous training points). For example, SMI for the *j*th input candidate is defined below.

(8) $$\begin{array}{ccc} smi(x_{j}^{t}) & = & I\left(x_{j},x_{j}^{t}\right)+I\left(x_{2},x_{j}^{t}\right)\cdots +I\left(x_{n},x_{j}^{t}\right)\\ & = & \sum_{i=1}^{n}I\left(x_{i},x_{j}^{t}\right) \end{array}$$

This contains a correlation between the current point $x_{j}$ and all previous inputs. This smi(·) metric is accumulated as $SMI\left(X^{t}\right)=\left\{smi\left(x_{1}^{t}\right),\cdots smi\left(x_{m}^{t}\right)\right\}$ and we picked the best one that was the least correlated with previous inputs (9). For updating the Gram matrix, we updated the Gram submatrix $K_{X^{t}X}$ as in Algorithm 1 (line 5).

(9) $$x_{best}^{t}=\underset{x}{\mathrm{argmin}}SMI\left(X^{t}\right)$$

**Figure 3:**
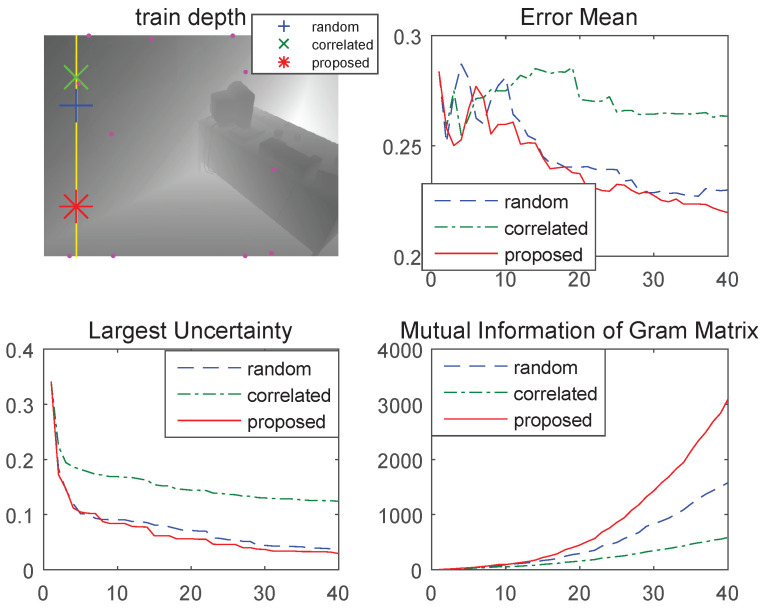
Depth estimation comparison with three different point selection criteria. (Left top) Previous inputs (magenta, dot) and three types of selected points. Each step is a line of the input candidates (yellow line) obtained. In the three performance graphs, the proposed point (red, solid) shows better results than random (blue, dashed) and correlated (green, dash-dotted).

We compared the quality of three possible candidates in Figure 2 and Figure 3. From the SMI bar in Figure 2, we selected three types of selecting criteria (proposed, randomly, and mutually related). Figure 3 describes the toy example results. In this validation, we assumed depth information was given as a vertical line (yellow line on the image) for each time step. Among the raw inputs for selecting the candidates, we chose the best one according to three criteria—error mean, maximum uncertainty, and MI of *K*. The graph shows depth estimation when the selecting criteria differ. Using the proposed point selection, the average error drops faster, largest uncertainty is lower, and MI increases faster than the other two methods (random, correlated).
**Algorithm 1** Active point(s) selection1:**Input:** Input image $I_{in}$, Previous inputs X, New inputs $X^{t}$, Step *T*2:($K_{X^{t}X},k_{x_{1}^{t}},k_{x_{1}^{1}})\leftarrow$Compute-K(X, $X^{t}$)3:$SMI(X^{t})\leftarrow$Compute-SMI($K_{X^{t}X},k_{x_{1}^{t}},k_{x_{1}^{1}})\;$▹(8)4:$x_{best}^{t}={\mathrm{argmin}}_{x}SMI\left(X^{t}\right)\;$▹(9)5:$K_{X^{t}X}\leftarrow$Update-K($K_{X^{t}X},x_{best}^{t}$)6:**Output:** Gram submatrix $K_{X^{t}X}$, Selected input $x_{best}^{t}$

### 4.2. Stop Criterion

The more training data we used, the better the GP regression results. However, memory and computational issues existed as the training set increased. Therefore, we also propose a stop criterion for training based upon the information of the Gram matrix. A similar approach was presented in [26], who used MI ($I_{K}=\left|I+\sigma^{-2}K\right|$) for the GP optimization. Contal et al. [26] introduced upper bounds on the cumulative regret by an MI-based quantity measure. Motivated by this, we reversely utilized the increment of MI information to decide the quality of training inputs and a GP model. We computed a similar metric but used $K_{G}=K+\sigma^{2}I$.

(10) $$I_{K_{G}}=\left|I+\sigma^{-2}K_{G}\right|$$

We computed the delta between two consecutive time steps, *t* and t−1, by calculating $\Delta I^{t}=I_{K_{G}}^{t}/I_{K_{G}}^{t-1}$. By comparing $\Delta I^{t}$ and $\Delta I^{t-1}$, a stop flag was activated if the increment amount of step *t* dropped under a particular rate (α) of the previous step (i.e., t−1, $\Delta I^{t}<\alpha \Delta I^{t-1}$). We used α=0.80 and stop training if the increase of information was slower than 80% of the previous update. The detailed procedure of dense depth map estimation with informative point selection is described in Algorithm 2.
**Algorithm 2** Online Dehazing with iGP1:**Input:** Input image $I_{in}$, set of training inputs *X*, set of training outputs *Y*, number of point sets $t_{end}$2:t=1, $fl_{stop}=0$, X=[]3:**while** ($t\leq t_{end}$) || ($fl_{stop}==1$)4:  **if t==1**5:    $(K,X_{init})\leftarrow$Initialize-K($X^{t}$)6:    $X=[X,X_{init}]$7:    $K_{G}=K+\sigma^{2}I$8:    (Q,R)←QR-Decompse($K_{G}$)9:    $K_{G}=R$10:    t=t+111:  **else**12:    $(K_{X^{t}X},x_{best}^{t})\leftarrow$ActivePointSelect($I_{in},X,X^{t}$)13:                                          ▹ Algorithm 114:    $n_{skip}=size\left(K\right)$15:    $K_{pre}=K_{G}$16:    $K_{G}=\left[K_{G},K_{X^{t}X}^{⊤};K_{X^{t}X},K_{X^{t}X^{t}}+\sigma^{2}I\right]$17:    (Q,R)←QR-Decompse($K_{G},Q,n_{skip}$)18:    $K_{G}=R$19:    t=t+120:    $X=[X,x_{best}^{t}]$21:    $fl_{stop}\leftarrow$StopCriterion($K_{G},K_{pre}$)                       ▹Section 4.222:  **end if**23:**end while**24:$K_{G}^{-1}=R\backslash Q^{⊤}$25:$X_{*}\leftarrow$MatToArray($I_{in}$)26:$K_{X_{*}X}\leftarrow$Compute-K($X_{*},X$)27:$D_{map}\leftarrow$ComputeGP-Mean($K_{X_{*}X},K_{G}^{-1},Y$)28:**Output:** Dense depth map $D_{map}$

### 4.3. Dense Depth Map-Based Haze Removal

Having predicted the dense depth map, we could reconstruct the haze-free image via (1). Unknown parameters are airlight *A* and attenuation coefficients β. First, *A* can be estimated from the depth map and an input image motivated from [8]. The dense depth map was already prepared; therefore, we picked pixels from 0.1% of the deepest points in the depth map and selected the pixel color that had the maximum brightness. These pixel color values were used for the *A* input image. Moreover, we estimated β by fitting the transmission model with brightness decaying nearest ($i_{min}$) and the deepest ($i_{max}$) pixels. In other words, the estimated β is $\beta =i_{max}/i_{min}$. The last part of the image dehazing is white balance. It is not effective for a normal aerial image with airlight near white; however, it helps restore biased color for underwater and indoor environments. The dehazing algorithm is described in Algorithm 3. It takes input image $I_{in}$ and depth map $D_{map}$, and returns dehazed image $I_{out}$.
**Algorithm 3** Image Dehaze1:**Input:** Input image $I_{in}$, dense depth map $D_{map}$2:A←EstimateArilight($I_{in}$, $D_{map}$)3:$(A,i_{max},i_{min})\leftarrow$EstimateArilight($I_{in}$, $D_{map}$)4:β←EstimateBeta($i_{max},i_{min}$)5:**Output: $I_{out}\leftarrow$**ApplyDehazing($I_{in}$, *A*, β)

## 5. Experimental Results

In this section, we conducted experiments on both synthetic and real-world datasets to evaluate the proposed method. We compare the proposed method to the well-known approaches, including model-based methods (References [7,12,28]), learning-based methods (References [21,22,29,30]), and underwater image enhancement methods (References [13,16,18,19,30,31,32]). In addition, we evaluated our method on grayscale images and for its performance in geometric vision applications, such as the feature detection of the processing time.

### 5.1. Datasets

We first constructed 10 synthetic haze images using well-known RGB-D image datasets (ICL-NUIM [33], which contained sequential image frames with ground-truth camera motion. Moreover, we captured real hazy images in the indoor environment using an artificial fog generator. Each dataset included 20 hazy images with ground-truth and real hazy images. We also tested the proposed method on various image degradation scenarios to evaluate it thoroughly. We implemented the proposed method to the dehazing benchmark datasets, such as HazeRD [34] and O-Haze [35] datasets. Each HazeRD and O-haze dataset contained 14 synthetic hazy images and 45 real outdoor hazy images with their corresponding ground-truth images. Moreover, we tested the proposed method on the 20 well-known hazy/foggy images utilized in the various dehazing research studies [7,21,30]. For underwater hazy images, we used famous underwater test images utilized from previous research studies. We selected 20 underwater images to extract image quality metrics.

Each dataset contained various hazy images with different characteristics, we categorized the datasets as follows:

- Synthetic haze images with ground-truth;
- Real indoor and outdoor haze images;
- Grayscale haze images;
- Real underwater images without ground-truth.

### 5.2. Implementation Details

The enhancement algorithm was written in MATLAB, and the test was performed on Intel i7-9750H CPU@2.60 GHz, 16 GB RAM. For datasets that included real depth information, we used a row-wise slice of the depth as the given measurement and our method automatically selected 10 training depth points to construct the Gram matrix. If the datasets did not have ground-truth depth information, we constructed a rough depth map and used it to estimate the transmission of the haze model. All of the compared results were obtained from the authors’ provided code and tested on the same system. To test the performance of deep learning-based methods, we also utilized an RTX3090 GPU. Additionally, we included the original and used image sizes of each method in the every figure for qualitative evaluations, as the performances of the compared algorithms depended on the input image size.

### 5.3. Evaluation Metrics

To compare the results of our proposed method with previous approaches, we used both supervised and unsupervised image quality metrics for aerial and underwater images. We measured the performance of image restoration using similarity-based metrics, such as the structural similarity index (SSIM) and peak signal-to-noise ratio (PSNR). Additionally, we examined edge-level metrics, such as level [36] and *r* [37], which indicate medium-scale edges and visible edges after enhancement. For underwater images, we employed well-known blind image quality metrics: Underwater Image Quality Measures (UIQM) [38] and Underwater Color Image Quality Evaluation (UCIQE) [39]. To facilitate visualization, we highlighted the best and second best results in red and blue font in Table 1, Table 2 and Table 3, respectively. For better visualization in the result tables, we marked the best result in red and the second best one in blue.

PSNR is used for evaluating the loss on image quality, i.e., the lower the loss, the higher the value. MSE, which is a molecular part of the PSNR equation, calculates a difference in pixel intensity between two images. In the MSE equation, MN means the number of image pixels and *I* represents pixel intensity. When MSE is 0, the images should be the same. *R*, which is the denominator portion, means the maximum value of the pixel. It is 255 for ’uint8’ (8-bit unsigned integer), and 1 for the double or float type. Since PSNR is not a method used in expressing the difference in quality that humans visually perceive, even if the value is high, the image quality with a smaller value may look better from human eyes.

(11) $$MSE=\sum_{M,N}\frac{{\left[I_{1}\left(m,n\right)-I_{2}\left(m,n\right)\right]}^{2}}{MN}$$

(12) $$PSNR=10log_{10}\frac{R^{2}}{MSE}$$

Unlike PSNR, SSIM is designed to assess the visual quality rather than numerical differences. SSIM assesses quality in terms of luminance, contrast, and structure. When L, the dynamic range value, is an 8-bit unsigned integer (uint8), it is set to 255. Luminance refers to the amount of brightness in light. It is calculated using the pixel values of an image and is represented by the average luminosity, denoted by μ. In contrast, it refers to the degree of variation in brightness within an image and it is quantified through the standard deviation σ, which is calculated using the difference in values between pixels. The structure represents a structural difference in pixel values. Checking the similarity of the structural components of the two images has the same meaning as using the correlation of the two images. The equation of structure solves this structural difference.

(13) $$SSIM\left(x,y\right)={\left[l\left(x,y\right)\right]}^{\alpha }*{\left[c\left(x,y\right)\right]}^{\beta }*{\left[s\left(x,y\right)\right]}^{\gamma }$$

(14) $$l\left(x,y\right)=\frac{2\mu_{x}\mu y+C_{1}}{\mu_{x}^{2}+\mu_{y}^{2}+C_{1}}$$

(15) $$c\left(x,y\right)=\frac{2\sigma_{x}\sigma_{y}+C_{2}}{\sigma_{x}^{2}+\sigma_{y}^{2}+C_{2}}$$

(16) $$s\left(x,y\right)=\frac{\sigma_{xy}+C_{3}}{\sigma_{x}\sigma_{y}+C_{3}}$$

where $C_{1,2,3}$ represent the stabilization constants for the weak denominator.

Moreover, we utilized edge-related metrics (level, *r*) to assess the quality of enhancement. The metric level [36] considers medium-scale gradients that are above a certain threshold and are not influenced by noise. The mathematical formulation is defined as follows:

(17) $$\begin{array}{c} \left\{\begin{array}{cc} \;\frac{1}{N}log\left(\lambda \left(m_{i}-\delta \right)+1\right) & \mathrm{if}\;m_{i}\geq \delta \\ \;0\; & \mathrm{otherwise} \end{array}\right. \end{array}$$

where $m_{i}$ is the gradient magnitude at pixel location *i*, δ is the activation threshold value, λ is a control parameter to adjust the total value, and $m_{i}$ represents the amount of gradient information corresponding to the gradient magnitude. *N* is a normalization factor to bound the output range of the function to [0,1]. Finally, we can calculate level, which is the total amount of gradient information in an image as in

(18) $$\begin{array}{ccc} level & = & \sum_{}{\bar{m}}_{i}. \end{array}$$

The metric *r* [37] represents the visible edges after image restoration. The mathematical form of the metric is defined as below.

(19) $$\begin{array}{ccc} r & = & exp\left(\frac{1}{n_{r}}\sum_{pi\epsilon \psi \bar{r}}log{\bar{r}}_{i}\right) \end{array}$$

where $n_{o}$ and $n_{r}$ denote the cardinal numbers of the set of visible edges in the original image ($I_{o}$) and the restored image ($I_{r}$).

For underwater images, we used unsupervised image quality metrics UIQM and UCIQE. First, UIQM [38] is used to show the colorfulness, sharpness, and contrast measures of the restored images. UIQM is defined as below:

(20) $$\begin{array}{c} UIQM=c1*UICM+c2*UISM+c3*UIConM \end{array}$$

where UICM, UISM, and UIConM represent colorfulness, sharpness, and contrast with the pre-defined coefficients c1 = 0.0282, c2 = 0.2953, and c3 = 3.5753.

Motivated by human perception, UCIQE is designed to validate the quality of images with the classic distortion types of data acquired in turbid water, involving a linear combination of chroma, saturation, and contrast; it is proposed to quantify non-uniform color cast, blurring, and low contrast. The following equation represents the mathematical formula of the metric.

(21) $$UCIQE=c_{1}\times \sigma_{c}+c_{2}\times con_{l}+c_{3}\times \mu_{s},$$

where $\sigma_{c}$ is the standard deviation of chroma, $con_{l}$ is the contrast of luminance, $\mu_{s}$ is the average of saturation, and each *c* means the weighted coefficients. The variance of chroma is deeply related to the perception of the part that humans are interested in.

### 5.4. Synthetic Haze with Ground-Truth

**Figure 4:**
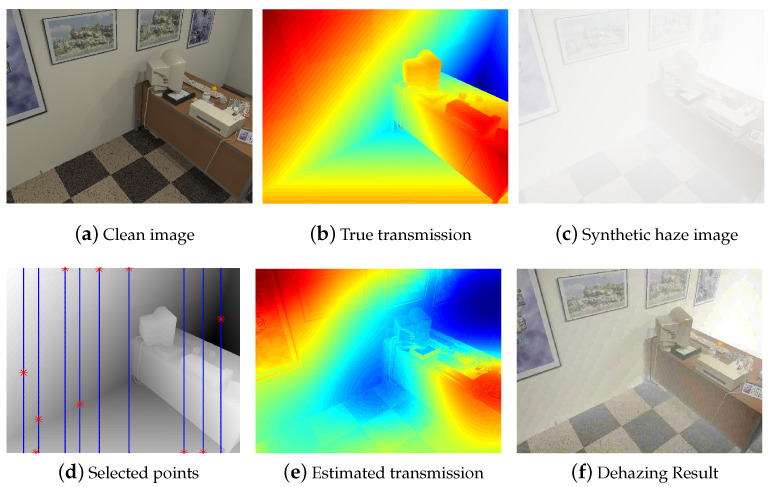
Dehazing example of the synthetic haze image. (**a**) Original clean image of ICL-NUIM [33]. (**b**) True transmission map (red: high, blue: low). (**c**) Synthetic haze image by adding artificial fog from the transmission map. (**d**) Selected points chosen by the SMI (red star) given the candidate points (blue dot) of each update. (**e**) Estimated Transmission map. (**f**) Image dehazing result.

In this section, we first evaluated the proposed method for synthetic hazy images with ground-truth. We utilized HazeRD [34] dehazing benchmark datasets, which included 14 hazy images with ground-truth depths and clean images. Moreover, we constructed a new synthetic haze dataset via a publicly available ICL-NUIM RGB-D [33] benchmark dataset as represented in Figure 4. The ICL-NUIM RGBD benchmark dataset offers a full depth map without the missing depths of all pixels; therefore, we could synthetically corrupt original images (Figure 4a) with different fog levels (Figure 4c). We applied a synthetic haze on haze-free images by (1) using a transmission map (Figure 4b) computed from a true depth map. For the sparse depth assumption, we sampled 10 vertical lines of depth points as candidates and selected only one best point for each updated line of depth.

Figure 4 describes the dataset with the synthetic haze and dehazing processes of the proposed method. As represented in Figure 4d, the proposed method automatically samples and selects the best training points (red stars), as it is given hundreds of input depth points (blue dots). Figure 4e depicts the transmission estimation and dehazing results compared to the other methods. Using 10 training inputs, we estimate a reliable transmission map and a haze-free image.

**Figure 5:**
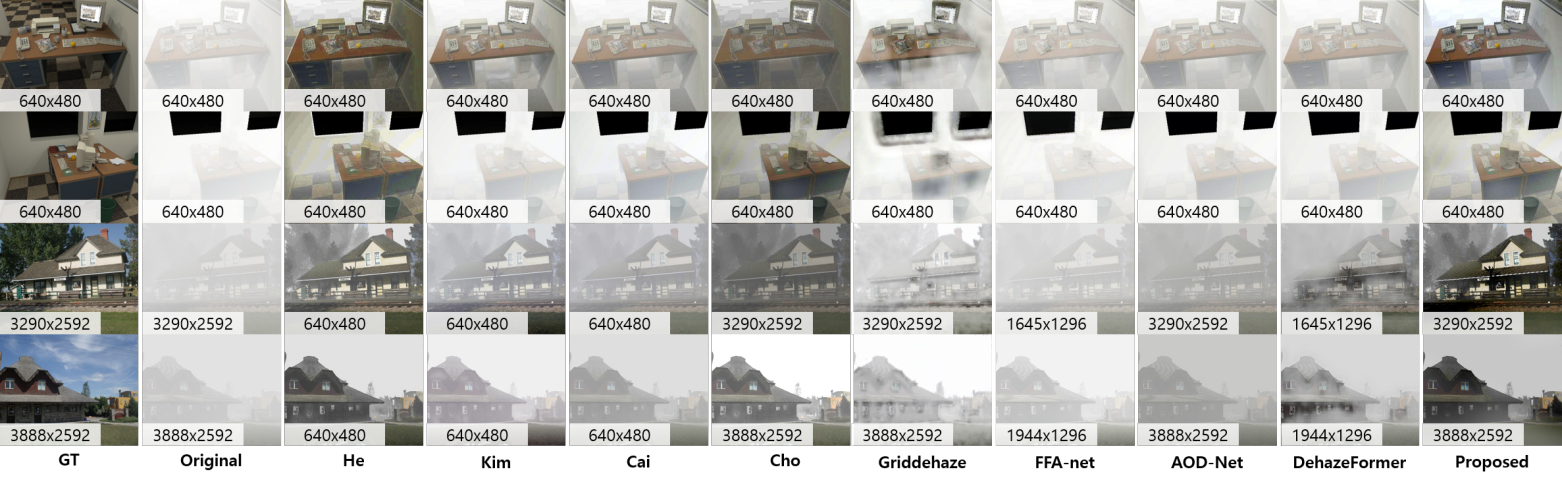
Image dehazing results on the synthetic hazy images with ground-truth. We evaluated the proposed method compared to model-based methods (He [7], Cho [12], Kim [28]) and Learning-based methods (Cai [30], AOD-Net [20], Griddehaze [22], FFA-Net [29], and DehazeFormer [21]).

Figure 5 represents the qualitative comparisons of the proposed method and previous research studies: model-based approaches (He [7], Cho [12], Kim [28]) and Learning-based methods (Cai [30], AOD-Net [20], Griddehaze [22], FFA-net [29], and DehazeFormer [21]). The image size is mentioned at the bottom of each figure. Generally, the model-based approaches show better performances than learning-based approaches. Because the learning-based methods highly depend on the characteristics of training datasets, the deep networks show limitations in covering dense, hazy images, such as the test images. For example, Griddehaze and DehazeFormer showed block pattern artifacts because of the grid assumption and sequential link of the networks. Cai and AOD-Net showed relatively stable results; however, the dehazing performance was not enough to restore the original pixel intensities. Among the model-based approaches, the proposed method yields the best performance. Moreover, this result is represented in Table 1. We utilized quantitative image metrics that described restoration quality (PSNR and SSIM) and edge-level enhancement quality (level and *r*). As described in the table, the proposed method performed best considering all evaluation metrics.

**Table 1:** Quantitative evaluation of synthetic hazy images represented in Figure 5. The metric values are computed by average scores of 14 images of HazeRD [34] and 10 synthetic images from [33] including the images in the figure. The best and second best performances are described in the red and blue texts.

Data	PSNR ↑	SSIM ↑	level ↑	*r* ↑
He [7]	18.598	0.537	0.554	1.402
Kim [28]	10.820	0.380	0.450	1.413
Cai [30]	12.61	0.357	0.448	0.975
Cho [30]	11.732	0.431	0.542	1.681
Griddehaze [22]	12.55	0.374	0.448	1.826
FFA-Net [29]	11.154	0.330	0.416	1.818
AOD-Net [20]	11.342	0.366	0.527	1.422
DehazeFormer [21]	12.63	0.372	0.529	1.512
Proposed	19.945	0.567	0.590	2.152

### 5.5. Real Indoor/Outdoor Haze

**Figure 6:**
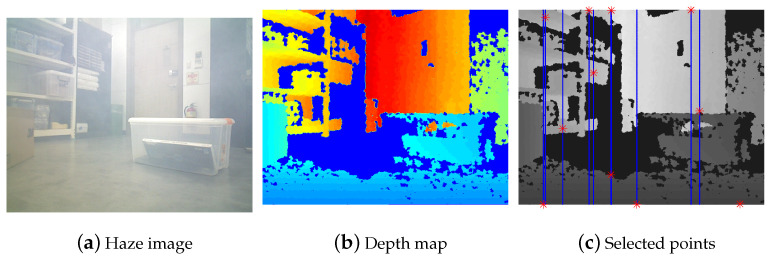
(**a**) Haze image. (**b**) Depth map from RGBD sensor (red: far, blue:near). (**c**) Candidate points (blue, dot) and selected point (red, star).

Additionally, we tested the proposed method with real indoor and outdoor hazy images. We first evaluated the proposed method on 45 dehazing benchmark images from the O-haze [35] dataset. Moreover, we validated the proposed method on several well-known test images used in previous research studies [7,28,30]. Then, we tested our approach on real indoor hazy images. By using an RGB-D sensor and an artificial fog machine, we constructed datasets that included 30 real hazy images with the depth sensing information. The sample image and depth map are described in Figure 6.

**Figure 7:**
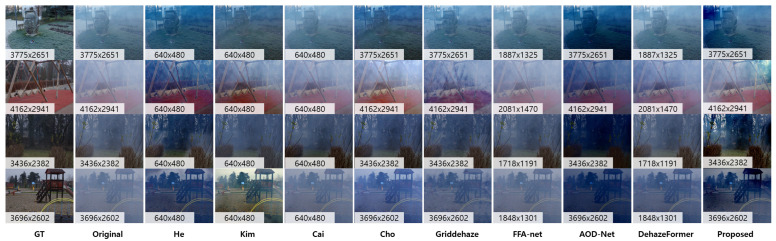
Image dehazing results on the real in/outdoor datasets without ground-truth. We evaluated the proposed method compared to the model-based methods (He [7], Kim [28]), and learning-based methods (Cai [30], AOD-Net [20], Griddehaze [22], FFA-Net [29], and DehazeFormer [21]).

As mentioned, we first evaluated all of the test methods using the O-haze [35] benchmark dataset. Figure 7 shows the results of the qualitative evaluation by comparing other physics-based methods and deep learning-based methods with the proposed method. As shown in Figure 7, which includes ground-truth images, He et al. [7] used DCP to estimate transmission; this priority was invalid when objects were similar to airlight. In addition, this model was optimized for air haze images with a wide range of transmission differences. Kim et al. [28] created repetitive spotted artifacts from the center of the image to the outside. Moreover, the results show that our model performs better when compared with deep learning methods. Griddehaze-net [22] eliminated hazing features irregularly. Dehazeformer [21] and AOD-Net [20] resulted in similar performances. Cai et al. [30] performed well but it had low contrast (more than the proposed method).

We also compared the performance quantitatively. Table 2 presents the corresponding performance from Figure 7. As in the table, the proposed method outperformed the evaluation metrics. While He [7] showed the highest scores on SSIM and *r* metrics, the proposed method has almost similar performances to the best scores.

**Table 2:** Quantitative evaluation on O-haze datasets [35] represented in Figure 7. The metric values were computed by average scores of 45 images of O-haze datasets including the images in the figure. The best and second best performances are described in the red and blue texts.

Data	PSNR ↑	SSIM ↑	level ↑	*r* ↑
He [7]	22.012	0.600	0.156	1.165
Kim [28]	16.814	0.514	0.089	0.891
Cai [30]	14.95	0.458	0.135	0.975
Cho [12]	16.49	0.553	0.144	0.987
Griddehaze [22]	12.68	0.429	0.162	0.932
FFA-Net [29]	11.45	0.369	0.105	0.524
AOD-Net [20]	12.17	0.382	0.158	0.648
DehazeFormer [21]	11.34	0.366	0.126	0.648
Proposed	24.302	0.588	0.170	1.011

**Figure 8:**
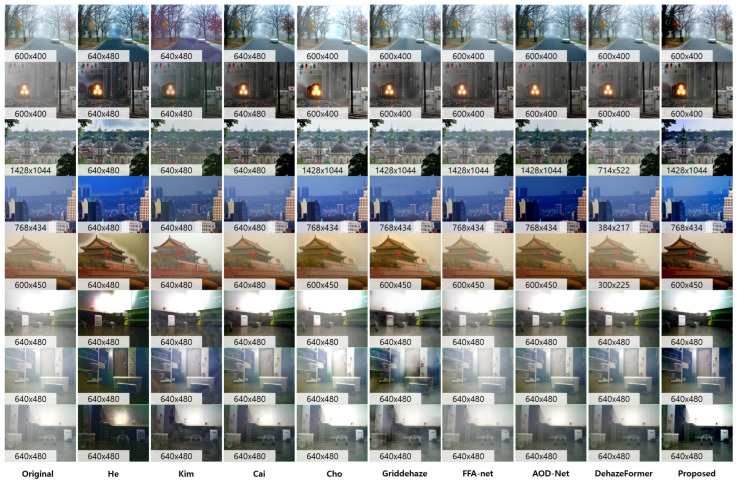
Image dehazing results on synthetic haze and real indoor hazy scenes. We evaluated the proposed method compared to model-based methods (He [7], Kim [28]), and learning-based methods (Cai [30], AOD-Net [20], Griddehaze [22], FFA-Net [29], and DehazeFormer [21]).

In addition, we evaluated the performances to real in/outdoor sequences without ground-truth as in Figure 8. We chose several well-known hazy scenes from previous research studies (first–fifth row). Moreover, we captured real indoor hazy scenes using artificial fog machines (sixth-eighth row). The proposed method is generally well-dehazed, and the image’s contrast is adequately maintained so that nearby objects and distant objects are clearly visible. For images up to the fourth and seventh rows, AOD-net Li et al. [20] makes it difficult to recognize objects by dehazing in a way that attempts to increase the contrast too much. The learning-based methods [20,21,22,29,30] showed better performances compared to the previous evaluations, see Figure 5 and Figure 7. However, the performance was not enough to enhance contrast and visibility compared to the proposed method. Note that we utilized the networks with the trained weights given by the authors.

### 5.6. Real Indoor Haze (Gray Images)

We also validated with grayscale images. As known from the literature, it is challenging to dehaze gray images since they only have single channels. For the evaluation, we compared our method with the methods that could be applied to the single channel images, such as He et al. [7] and Tarel and Hautiere [37].

**Figure 9:**
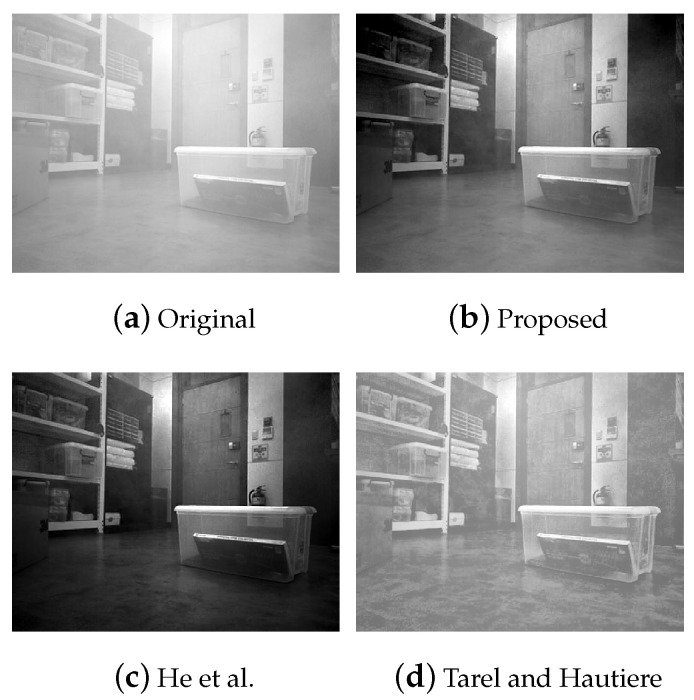
Dehazing result of gray scale images under artificial fog. (**a**) Input haze image. (**b**) Proposed method. (**c**) Result of He’s method [7]. (**d**) Result of Tarel’s method [37].

Figure 9 depicts the dehazing result of the gray indoor image. Our method showed good dehazing results without intensity distortion. In comparison with our reliable results, the intensity of [7] was shifted lower. Therefore, the brightness of the result figure was distorted. This is because DCP used minimum color relations between patches. On the other hand, the brightness of the result of [37] was similar to the input haze image. However, the texture of the floor and objects in the input image were transformed.

### 5.7. Real Underwater Scene

**Figure 10:**
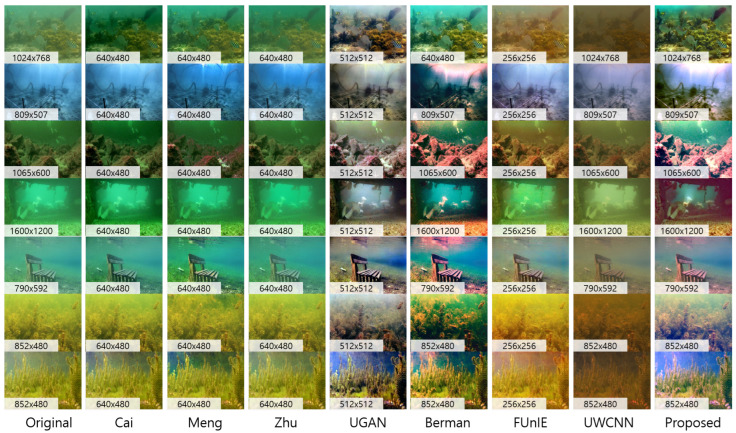
Enhancement results of the underwater images. We evaluated the proposed method compared to model-based methods (Cai [30], Meng [31], Zhu [32]), and learning-based methods (UGAN [18], Berman [13], FUnIE [19], and UWCNN [16]).

In this section, we evaluate the proposed method on real underwater images as described in Figure 10. Test images were selected from the previous underwater image enhancement research studies and collected from Google to test the methods in various environments (global veiling light color and objects). As mentioned, we assumed sparse depth priors for dehazing. In underwater environments, we cannot directly use depth measurements from images. For this reason, we utilized a rough depth map without any details of the images. These depth maps contained only relative range information (near or far) without details.

Moreover, the proposed method resulted in contrast-enhanced and color-balanced images compared to previous methods. Dehazing results of underwater color images are described in Figure 10. As seen in this figure, the proposed method showed the best performance results only with sparse depth priors independent of background colors (bluish, greenish, and yellowish). Due to the color distortion and bias of underwater images, Cai [30], Meng [31], and Zhu’s method [8] mispredicted both transmission and global veiling light. Although we aimed for online image dehazing without GPU usage, we also tested the proposed method to the recent learning-based methods ([16,18,19]) for a fair comparison. References [16,19] did not perform well in various water environments because of the dependency of training datasets. Rather than [18], it can be seen that near objects appear reddish (third and fourth rows) because of the color shift effect of the Lab correction using the Flickr dataset. Nevertheless, the visibility of ours was further secured in the first and fourth rows. The result of [13] appeared more reddish than the proposed model, and the overall color tended to be mapped to a darker domain.

In the underwater domain, Table 3 showed that ours represented the second-best performance evaluated with UIQM and UCIQE metrics. Even this showed almost the same performance as UGAN [18], which showed the best score. Considering the computation efficiency, our method shows better efficiency because UGAN is a heavy network with approximately 54M parameters and needs a high-performing GPU for processing.

**Table 3:** Quantitative evaluation of the underwater image.

Data	UIQM [38] ↑	UCIQE [39] ↑
Cai [30]	0.4412	23.1243
Meng [31]	0.5361	23.8983
Zhu [32]	0.4361	19.9227
UGAN [18]	1.0351	31.0920
Berman [13]	0.9015	31.5963
FUnIE [19]	0.4758	23.1054
UWCNN [16]	0.3934	15.3755
Proposed	0.9989	29.1589

### 5.8. Feature Detection and Processing Time

**Figure 11:**
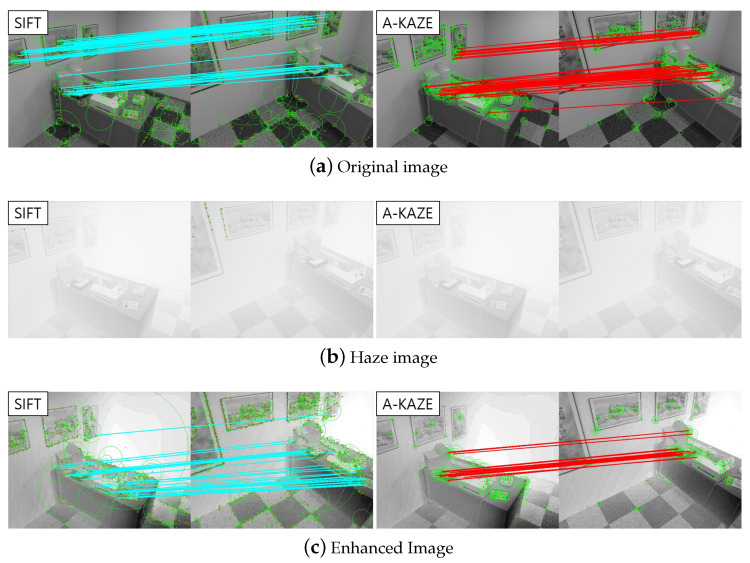
Feature (SIFT and A-KAZE)Detection Results. Extracted keypoints (green circle) and corresponding matching points (cyan line, red line) are shown. (**a**) Original haze-free images. (**b**) Hazy images. (**c**) Dehazed image from our method.

Lastly, we evaluated our method via feature detection results. The Scale Invariant Feature Transform (SIFT) feature proposed by Lowe [40] and KAZE feature proposed by Alcantarilla et al. [41] were used for feature extraction and matching. SIFT reported stronger matching results as can be seen in Figure 11. Compared with the original haze-free image of the synthetic dataset, the detection results of haze and dehazed images are shown in Figure 11. The quantitative comparison result is in Table 4. From Figure 11c and Table 4, feature detection results are much better in dehazed images. Although the number of inliers slightly decreased, the inlier ratio was higher than the original. This aspect seemed to be achieved from the fact that small textures became smooth and strength feature points were restored after dehazing. Other quantitative comparisons were also provided in Table 5. We tested the computation time of the proposed method compared to conventional model-based approaches [6,7,8,9,37]. The test image size was fixed to the VGA (640 × 480) size. As reported in the table, the proposed method showed efficiency on the computation.

## 6. Conclusions

This paper focused on iGP-based real-time haze removal with sparse partial depth cues. We first set the kernel function as a mixture of SE and NN kernels for better estimation performances on real datasets with sudden spatial changes. Moreover, a new SMI metric was introduced for selecting the best points among newly added inputs. With this information measure, we can avoid unnecessary training and keep the estimation model efficient; thus, the algorithm can operate in real time. Having obtained a depth map, in addition, we estimated parameters (*A*, β) in a dehazing model that were chosen automatically. For evaluation, we tested the method on synthetic fog and real indoor haze datasets (for color and gray images). In addition, we evaluated the proposed method to underwater images.

There were a few problems, however, which need real applications. First, dehazing parameter estimation is a rough approximation. In our experiments, this method is enough to predict reasonable parameter values. However, it is necessary to test this part in various environments. The second part involves constructing a global GP model for robust estimation using sequential and multiple input images.

## Figures and Tables

**Table 4:** Feature extraction result.

	Num. of Matches		Num. of Inliers		Inlier Ratio	
	**SIFT**	**KAZE**	**SIFT**	**KAZE**	**SIFT**	**KAZE**
Clean	142	130	45	41	31.69 %	31.53 %
Haze	0	5	0	0	0	0
Proposed	88	82	30	30	34.09 %	36.58 %

**Table 5:** Computation time comparison (s).

Data	Proposed	[6]	[9]	[7]	[8]	[37]
syn1	0.5648	63.0368	75.1875	27.1568	1.2028	-
indoor1	0.5947	65.9826	78.7996	26.7472	1.5637	-
indoor gray	0.5348	-	-	24.5654	-	8.5041

The methods of [6,8,9] are excluded from the test on gray images due to dependency of the channel number. The method of [37] was tested on gray images only since it does not estimate transmission.

## Data Availability

Not applicable.
